# Analysis of human mini-exome sequencing data from Genetic Analysis Workshop 17 using a Bayesian hierarchical mixture model

**DOI:** 10.1186/1753-6561-5-S9-S93

**Published:** 2011-11-29

**Authors:** Julio S Bueno Filho, Gota Morota, Quoc Tran, Matthew J Maenner, Lina M Vera-Cala, Corinne D Engelman, Kristin J Meyers

**Affiliations:** 1 Department of Dairy Science, University of Wisconsin–Madison, 444 Animal Science Building, 1675 Observatory Drive, Madison, WI 53706-1284, USA; 2 Departamento de Ciências Exatas, Universidade Federal de Lavras, PO Box 3037, Lavras, MG 37200-000, Brazil; 3 Department of Statistics, University of Wisconsin–Madison, 1300 University Avenue, Madison, WI 53706, USA; 4 Department of Population Health Sciences, University of Wisconsin–Madison, 707 WARF Building, 610 North Walnut Street, Madison, WI 53726, USA; 5 Departamento de Salud Pública Universidad Industrial de Santander, Carrera 32 #29-31 Piso 3, Bucaramanga, Santander 680002, Colombia

## Abstract

Next-generation sequencing technologies are rapidly changing the field of genetic epidemiology and enabling exploration of the full allele frequency spectrum underlying complex diseases. Although sequencing technologies have shifted our focus toward rare genetic variants, statistical methods traditionally used in genetic association studies are inadequate for estimating effects of low minor allele frequency variants. Four our study we use the Genetic Analysis Workshop 17 data from 697 unrelated individuals (genotypes for 24,487 autosomal variants from 3,205 genes). We apply a Bayesian hierarchical mixture model to identify genes associated with a simulated binary phenotype using a transformed genotype design matrix weighted by allele frequencies. A Metropolis Hasting algorithm is used to jointly sample each indicator variable and additive genetic effect pair from its conditional posterior distribution, and remaining parameters are sampled by Gibbs sampling. This method identified 58 genes with a posterior probability greater than 0.8 for being associated with the phenotype. One of these 58 genes, *PIK3C2B* was correctly identified as being associated with affected status based on the simulation process. This project demonstrates the utility of Bayesian hierarchical mixture models using a transformed genotype matrix to detect genes containing rare and common variants associated with a binary phenotype.

## Background

The past decade of human genetics research has been dominated by Genome-Wide Association Studies (GWAS) and the common disease/common variant hypothesis. Although GWAS have successfully identified numerous single-nucleotide polymorphisms (SNPs) associated with common diseases, a large portion of the heritability for most diseases remains unexplained [1]. One proposed source of the missing heritability is rare variants. Rare variants (minor allele frequency [MAF] < 5%) are estimated to make up 60% of variation found in the human genome [2]. In addition to being abundant, these rare SNPs are more likely to have functional implications [2]. A new generation of genome sequencing technology, combined with a paradigm shift recognizing the importance of low-MAF SNPs, has led to the emergence of sequencing studies of the whole genome, whole exome, or targeted genes [2,3].

The prevailing statistical approach for estimating genetic effects in GWAS has been to test one SNP at a time for association with the phenotype of interest using linear or logistic regression. This approach is limited in sequencing studies because, by definition, sequencing studies identify rare genetic variants that individually do not provide statistical power for detecting associations. To address power limitations of individually rare variants, researchers have proposed numerous methods for pooling rare variants together within a predefined functional unit, often a gene [4]. Although these pooling methods increase statistical power for implicating a gene or genomic region, they are limited because they cannot determine which of the pooled SNPs has causal potential and because they ignore complexity by modeling only one gene at a time.

Bayesian hierarchical methods provide an alternative statistical approach for examining genetic sequence data [5]. These methods have several advantages over single-marker regression methods[5][6][7]. Bayesian methods provide the ability to specify prior layers of hierarchical structure as parameter dependencies (i.e., SNPs nested within genes). Another advantage of Bayesian methods is the simultaneous estimation of genetic effects, as opposed to regression methods that estimate the effect of each genetic variant independent of any other genetic markers. The purpose of this study is to use the Genetic Analysis Workshop 17 (GAW17) mini-exome sequence data to test the application of a Bayesian hierarchical mixture model for identifying genes containing rare and common variants associated with a simulated binomial outcome.

## Methods

### Data

This study includes 697 unrelated individuals from replicate 1 of the GAW17 data set. Genetic sequence data were provided by the pilot3 study of the 1000 Genomes Project and included 24,487 autosomal SNPs from the exons of 3,205 genes. Analysis was performed without any knowledge of the phenotype simulation process.

### Statistical model

We use a hierarchical Bayesian mixture model to identify genes associated with a dichotomous phenotype. The statistical model for each observation is:(1)

where *η_i_* is a linear combination of effects that represents the liability of disease (i.e., a logit transform of the probability of having the disease) for individual *i*, *y_i_* represents the outcome for individual *I*, and π*_i_* is the probability that individual *i* has the trait of interest. The model for the vector of liabilities is:(2)

where covariate effects (Age, Sex, Smoking) are adjusted through the vector *β* with design matrix *X*, the effects of SNPs within a functional region are represented by the vector *u* with design matrix Z^*^ (defined later), and *w* is a vector of indicator variables such that if *w_j_* = 0, then *u_j_* = 0, and if *w_j_* = 1, then ). Uniform prior distributions are assigned to nuisance parameters in *β*, and an independent inverse scaled chi-square prior distribution is used for .

We use two formulations of the SNP effects design matrix: *Z* and Z^*^. In both formulations, let *z_i_*_,_*_j_* be the genotype of individual *i* at variant *j*, and let the common allele *A_j_* and variant allele *a_j_* have frequencies .

We define elements of the *Z* matrix using the traditional additive genetic model: *z*(*a_j_a_j_*) = −1, *z*(*A_j_a_j_*) = 0, and *z*(*A_j_A_j_*) = 1. We use this *Z* formulation to detect linear dependencies within genes and thereby to identify SNPs for removal because of multicollinearity with at least one other SNP in that gene. To identify multicollinear SNPs, we add one SNP at a time into the matrix *K* and compute the determinant of the *K*′*K* matrix for each gene. If the determinant of the square matrix equals 0 after the addition of any SNP, this indicates collinearity with another SNP or set of SNPs already within the gene and that SNP is removed from *K*. We construct the final *Z* matrix by binding the resulting *K* matrices from each gene (*K*_gene1_, *K*_gene2_, …, *K*_gene3205_). This approach approximates haplotype analysis using a regression style of formulation with a minimal set of regressors. These models have a simpler structure than the usual variance component models for haplotype analysis, and the set of linearly independent SNPs within the gene potentially describes all haplotype variation.

In the second formulation of SNP effects, we define the elements of the Z^*^ matrix by:(3)(4)

and(5)

This Z^*^ matrix design brings standardizes the effects in *u* as if the population came from random mating and therefore provides greater leverage for detecting rare alleles as nonnull [8]. Additive effects for the subjects are the sum of the individual SNP effects in the liability scale, given by:(6)

and *u_j_* can be seen as a scaled average effect of a substitution of a common allele by a variant allele.

We assume equal variance across all genes and SNPs. Because the proportion of genes truly associated with disease status in the total data set is unknown, we treat a single mixing coefficient (*λ*, the proportion of associated genes) as a parameter to be estimated. We assign a *Β*(2, 18) prior distribution to *λ* such that *p*(*u_j_* = 0) = 1 − *λ* and . The prior distribution can and should be tailored to be appropriate for any given outcome being studied. We infer a gene’s importance in liability of disease on the distribution of the indicator variable in the posterior sample. This is an approximation of the marginal distribution for the probability of the gene carrying an associated variant (i.e., at least one SNP within that gene is associated with the outcome). Within genes, further inference can be made on which SNPs are more likely to be associated by investigating the posterior distribution of SNP effects within the gene.

We use a Metropolis Hasting algorithm to jointly sample each (*w_j_*, *u_j_*) pair from its conditional posterior distribution, and remaining parameters are sampled by Gibbs sampling. Four chains of 100,000 Markov chain Monte Carlo (MCMC) samples are drawn, and the first 50,000 samples are discarded as the burn-in period. The samples are thinned at a rate of 10, leaving 5,000 samples for inference. Convergence of Markov chains is confirmed using Raftery and Lewis diagnostics, as described in their studies [8,9]. We consider the final chain of 5,000 samples converged if its effective size is greater than 4,000. This implies low dependence in the final chain and low estimates of initial samples to discard. We also visually inspect the chain plot for any systematic trends. We calculate highest posterior density intervals for mixing parameters and for effects of SNPs.

Programs were implemented in R [10]. A detailed description of the sampling process can be found in the Appendix, and the R code is available to investigators upon request.

## Results

Of the 697 individuals, 209 were affected with the simulated dichotomous phenotype. Of the 24,487 SNPs included in the GAW17 data set, we removed 576 SNPs because of their collinearity with another SNP or combination of other SNPs in the same gene. This resulted in 23,961 SNPs for analysis, with at least 1 SNP from each of the 3,205 genes. The number of SNPs per gene varied from 1 to 203, with an average of 7.48. The MAF of SNPs varied from 0.000717 (private variants) to 0.50, with an average MAF of 0.0438.

Convergence of Markov chains was confirmed using Raftery and Lewis diagnostics, as described earlier. The posterior mode of *λ* was 0.0625 with a highest posterior density interval of [0.0059, 0.2320]. These values do not depart much from the *Β*(2, 18) prior distribution, and considering that 3,205 genes are analyzed, the number of genes contributing to the affected status can vary from 19 to 744.

**Figure 1:**
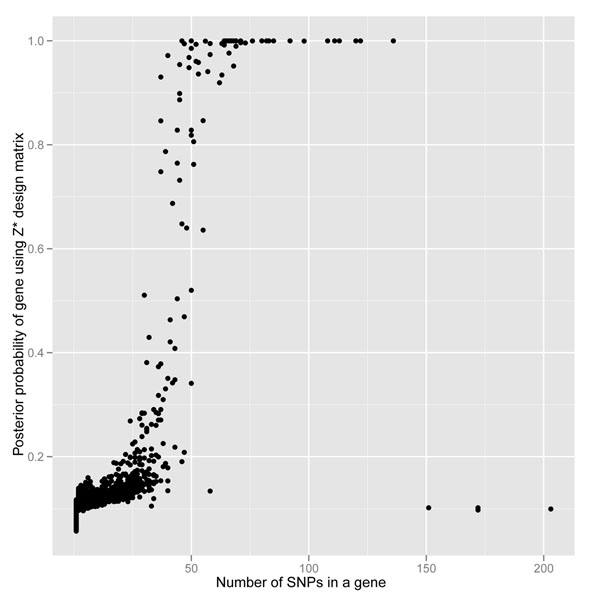
Scatterplot of posterior probability of a gene being associated with a dichotomous outcome of interest when using the allele frequency weighted Z^*^ design matrix. There were 58 genes with a probability greater than 0.80.

**Figure 2:**
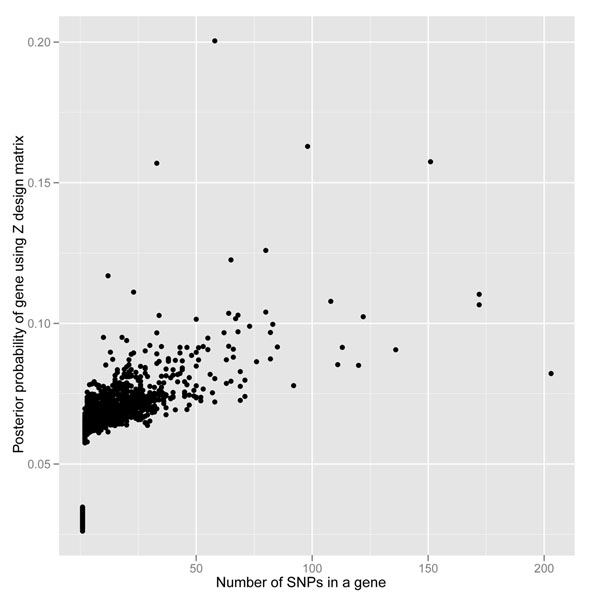
**Scatterplot of posterior probability of a gene being associated with a dichotomous outcome of interest when using the unweighted *Z* design matrix based on an additive genotype model.** There were no genes with a probability greater than 0.80.

We also computed the posterior probability of each gene being associated with the dichotomous outcome. Figure 1 is a scatterplot of the posterior probability for each of the 3,205 genes as a function of the number of SNPs within the gene when using the allele-frequency-weighted Z^*^ design matrix. As was done by Meuwissen and Goddard [11], we focused our inference on genes with a posterior probability greater than 0.8. Of the 3,205 genes, 58 had a posterior probability greater than 0.8 in at least one gene from each chromosome, and 23 had a posterior probability equal to 1 (Table 1). The mean MAFs (and standard deviations) were 0.0239 (0.072) for SNPs within the 58 genes and 0.0226 (0.069) for SNPs within the 23 genes. Out of the 58 genes with a posterior probability greater than 0.80, only *PIK3C2B* was designated as associated with the outcome in the simulation process. *PIK3C2B* had 71 SNPs, 24 of which were indicated as associated with the disease in the simulation. Based on our threshold of >0.80 for the posterior probability, our sensitivity and specificity for properly classifying genes as associated (or not associated) with the dichotomous outcome of interest were 0.028 and 0.982, respectively. Although we correctly identified only one of the associated genes, it is worth noting that not all SNPs within *PIK3C2B* were included in the simulation and that the SNPs that were included in the simulation had very low MAF (range, 0.000717–0.010760). We also ran the analysis using the unweighted *Z* design matrix. No gene was identified with a posterior probability greater than 0.20 (Figure 2).

**Table 1:** Characteristics of the 23 genes with a posterior probability equal to 1.0

Gene	Chromosome	Number of SNPs	Mean MAF
*PIK3C2B*	1	71	0.0270
*GOLGB1*	3	82	0.0210
*CENPE*	4	82	0.0165
*PCLKC*	5	65	0.0189
*PLEKHG4B*	5	80	0.0279
*BAT2*	6	83	0.0293
*UTRN*	6	111	0.0198
*RELN*	7	122	0.0147
*TG*	8	120	0.0103
*ANKRD15*	9	80	0.0284
*ERCC6*	10	64	0.0196
*TACC2*	10	108	0.0201
*SYTL2*	11	65	0.0417
*LRRK2*	12	67	0.0490
*POLE*	12	92	0.0173
*FREM2*	13	136	0.0173
*ALPK3*	15	68	0.0249
*VPS13C*	15	113	0.0160
*ABCC6*	16	66	0.0273
*BAIAP3*	16	50	0.0086
*HEATR6*	17	46	0.0054
*KIAA0802*	18	85	0.0195
*BRWD1*	21	69	0.0253

## Discussion

We have introduced a framework for the use of Bayesian hierarchical mixture models to identify genes associated with a dichotomous phenotype when those genes contain variants from across the entire allele frequency spectrum. Of particular interest in our method is the Z^*^ design matrix for leveraging rare variants and the subsequent ability of this model to estimate effects of SNPs and genes regardless of the MAF. This is a great advantage over the standard regression methods used in GWAS, which do not have the statistical power to detect SNP effects for rare or private variants. This method strongly resembles the stochastic search variable selection (SSVS) that has been used in quantitative trait locus analysis [6].

There are some limitations to our method. The most obvious is the high false discovery rate, a common theme of GAW17 and a known limitation in high-dimensional genetic association studies. After receiving the simulation answers from the GAW17 organizers, we identified a few potential sources for our high false discovery rate. One is the apparent mass effect bias, in which genes with more SNPs trend toward a higher posterior probability of being associated with the outcome (Figure 1). This bias arises because the fitting process comes from a conditional multiple regression and every regressor contributes to the probability of the gene. This fitting process could lead to false-positive results, and future extensions of this model should take gene size into account. Interestingly, the gene with the most SNPs, *AHNAK* (231 SNPs), was not identified with a posterior probability greater than 0.80. Therefore, although genes with larger numbers of SNPs trend toward increased posterior probability, this does not guarantee that the gene will be identified with a high probability of association (see bottom right-hand corner of Figure 1). Investigating different methods for assigning variances proposed in previous SSVS methods [6,12] may help to reduce this mass effect bias.

A second limitation of our method is the relative sensitivity to prior specification of the proportion of associated genes, *λ*. This is a common limitation of Bayesian hierarchical models that try to overcome the  problem. These models are sensitive to prior specification of the mixing parameter, at least with regard to the rate of convergence. Although our prior *β*(2, 18) achieved relatively quick convergence, the GAW17 simulation answers indicate that it was too large of a prior distribution and therefore contributed to our high false-positive rate. Future uses of this framework should consider using a more conservative prior distribution, although this will add to the computation time.

Our R code enabled gene-level inference after approximately 5 days of computing. We are currently working on improvements to lessen the already intensive computational time. A high-priority improvement in the method is a way to assess the acceptance of the gene in the model using a likelihood ratio test that allows for the most parsimonious model to be selected (i.e., some penalty for the number of parameters). An extension to accepting genes in the model would include a method for discarding SNPs from a selected gene. We could also consider including only nonsynonymous SNPs. However, we thought that this would be only a minor improvement because a good method should identify synonymous SNPs as noncausal (if they truly are). A final improvement would be to extend the hierarchy of the model to include the probability of a SNP being associated within a given associated gene. This probability could be estimated gene by gene and the final set of *w* variables could depend on both *λ*_1_ (gene being associated) and *λ*_2_ (SNP being associated in the *j*th gene).

We should also note that our high false discovery rate might be overestimated. Work done by another GAW17 group identified 695 genes that gave consistent false-positive results across numerous statistical methods and phenotype replicates [13]. From our list of 58 genes, 57 were incorrectly estimated to be associated with the binary phenotype, and 23 of these 57 were identified as consistent false positives by Luedtke et al. [13]. Although the reason that these genes give consistent false-positive results is still unknown, this issue highlights the importance of data quality control and the sensitivity of analytic methods to genotyping error or cryptic structure within the data.

Despite the limitations, our method has its advantages. Most notable is the ability of our method to more accurately represent genetic architecture through estimation of genetic effects conditioning on all other genetic effects and any risk factors of interest. Currently, most genetic association studies investigate one SNP at a time as though each SNP were independent of the others. Association studies using haplotypes attempt to better demonstrate dependency within the genome and functional units within genes. However, with sequence data, haplotype analysis will only worsen the dimensionality problems in association testing because of the number of rare variants. Our method of approximating haplotypes in a regression framework provides a more parsimonious approach than traditional haplotyping methods.

Another advantage of our method is the weighting of the design matrix by incorporating allele frequency information. Figures 1 and 2 compare the method when using the Z^*^ and *Z* genotype design matrices. No gene reached a posterior probability greater than 0.20 with the unweighted design matrix (*Z*); therefore the allele-frequency-weighted design matrix (Z^*^) greatly improves our leverage for detecting genes with a higher posterior probability of being associated with the outcome of interest.

## Conclusions

We applied a novel Bayesian hierarchical mixture model to sequence-level exome data for identifying genes and SNPs associated with a dichotomous phenotype. The analysis resulted in a substantial number of false-positive gene-level inferences, which appeared to be sensitive to the number of SNPs in each gene. Despite the high false discovery rate, we demonstrated a statistical approach that can simultaneously consider SNPs from the entire allele frequency spectrum. Further improvement of this approach, coupled with a growing understanding of sequence data, may contribute to advances in genetic epidemiological research.

## Competing interests

The authors have no competing interests to declare.

## Authors’ contributions

JSB and GM contributed to the conception, development, and execution of statistical analyses used in this manuscript, programmed the R code, and were integral to the writing and editing of the manuscript. QT, MJM, LMVC contributed to data manipulation, literature review, and general development of method. CDE supervised work on this project as part of the Genetic Analysis Workshop and provided feedback during method development. KJM oversaw development of this manuscript from conception through final publication through methodological consultation, coordinating collaboration among authors, assisting with data manipulation, and communication with the Genetic Analysis Workshop. All authors participated in the review, discussion, and critiquing of drafts of this manuscript.

## Appendix

The joint posterior distribution is given by:

in which *v* and *s*^2^ are the hyperparameters of the inverse scaled chi-square distribution for . Most of the parameters can be updated directly from their full conditional distributions:

where MVN stands for multivariate normal.

The following parameters require a tailored Metropolis Hastings step. For updating *λ*, use:

Next, sample λ^*^ from a candidate-generating function *q*(*c*, *d*) that is the same as the prior distribution, *Β*(*c* = 2, *d* = 18). Accept the outcome with probability:

For updating *w_j_* and *u_j_* (jointly), use:

in which:

and

Sample  from the candidate-generating function uniform(0, 1); if , then ; and if , then:

Accept  with probability:

For fast sampling, we took the average of the normal distribution from Eq. (A.10) instead of a random sample [9].
